# Impact of Hypothyroidism on Outcomes of Percutaneous Coronary Intervention with Coronary Atherectomy for Calcified Coronary Lesions: A Propensity-matched Analysis

**DOI:** 10.7759/cureus.2600

**Published:** 2018-05-09

**Authors:** Rupak Desai, Hemant Goyal, Gautam Kumar, Rajesh Sachdeva

**Affiliations:** 1 Research Fellow, Atlanta Veterans Affairs Medical Center, Decatur, Georgia; 2 Assistant Professor of Medicine, Department of Internal Medicine, Mercer University School of Medicine, Macon, Georgia; 3 Associate Professor of Medicine, Division of Cardiology, Emory University School of Medicine and Atlanta Veterans Affairs Medical Center, Atlanta, Georgia; 4 Associate Professor of Medicine, Division of Cardiology, Morehouse School of Medicine and Emory University School of Medicine, Atlanta, Georgia

**Keywords:** hypothyroidism, percutaneous coronary intervention, coronary atherectomy, calcified lesions, coronary atherosclerosis, all-cause mortality, outcomes, hospitalization cost, cardiovascular complications, pci

## Abstract

Overt and subclinical hypothyroid states have been depicted as risk factors for a more prominent level of coronary calcification. The utilization of coronary atherectomy with percutaneous coronary intervention (PCI) is on the ascent over the past few years as lesions that are more complex are now attempted with a percutaneous approach. However, the real-world data on hypothyroid status influencing PCI with coronary atherectomy outcomes is very limited. Therefore, we proposed to investigate the outcomes of percutaneous coronary intervention with coronary atherectomy in hypothyroid patients using the largest inpatient database in the United States (US), the National Inpatient Sample (NIS). To minimize the selection bias, we surveyed and compared the outcomes in both the unmatched and propensity-score matched euthyroid and hypothyroid cohorts. Concisely, this propensity-matched analysis, using the largest population-based sample in the US, has established higher cardiovascular comorbidities and worse clinical outcomes of PCI with coronary atherectomy owing to clinical hypothyroidism.

## Introduction

Overt and subclinical hypothyroidism have recently been recognized as the risk factors for a greater degree of coronary artery calcification [1, 2]. However, the real-world data on the clinical and subclinical hypothyroid status influencing the cardiovascular hemodynamics are limited. The impact of the hypothyroid state on the outcomes of percutaneous coronary interventions (PCI) specifically for the calcified coronary lesions subset have not been studied. There is a rise in the utilization of PCI with coronary atherectomy (CA) for complex lesions. Therefore, we decided to explore the outcomes of PCI with CA in hypothyroid patients using an inpatient database, the Nationwide Inpatient Sample (NIS).

## Materials and methods

The NIS is the largest publicly accessible all-payer inpatient database in the United States (US) and is sponsored by the Agency for Healthcare Research and Quality as a part of the Healthcare Cost and Utilization Project [3]. The discharge weights were applied to attain the national estimates, which minimizes the margin of error representing over 95% of the US population. After excluding patients <18 years of age, we identified patients in the years 2012-2014 who underwent PCI (00.66, 36.06, 36.07) with coronary atherectomy (CA) (17.55) using ICD-9 procedure codes. Patients with a diagnosis of hyperthyroidism (ICD-9 CM 242.x) and sick-euthyroid syndrome (ICD-9 CM 790.94) were excluded. Study subjects were divided into two cohorts: hypothyroid (ICD-9 CM code, 244.x) and a control cohort with remaining patients being considered as euthyroid. Chi-square test and independent sample t-test were performed to compare the baseline characteristics, outcomes (in-hospital mortality, length of stay (LOS), and hospitalization charges), and complications in unmatched and propensity-matched cohorts (1:1) adjusted for age, sex, race, median income, payer status, hospital characteristics, and relevant comorbidities. The primary outcomes were in-hospital mortality and postoperative complications, and secondary outcomes were the LOS and total hospital charges. Two-tailed p<0.05 was considered a threshold for clinical significance. IBM Statistical Package for the Social Sciences (SPSS) Statistics 22.0 (IBM Corp., Armonk, NY) was utilized to perform the analyses.

## Results

A total of 9,205 (weighted N=46,025) patients underwent PCI with CA of which 931 (weighted, N=4,655) were found to have hypothyroidism as a listed comorbidity and remaining 8,274 (weighted N=41,370) patients were considered as euthyroid. In unmatched comparison, hypothyroid patients were more frequently older (mean age 72±12 vs. 65±13, p<0.001), White (82.5% vs. 74.8%, p<0.001), females (54.6% vs. 27.3%, p<0.001) as compared to euthyroid patients. Interestingly, patients with hypothyroidism appeared to have higher rates of comorbidities such as hypertension (79.3% vs. 72.2%, p<0.001), diabetes with chronic complications (12.0% vs. 6.3%, p<0.001), dyslipidemia (73.4% vs. 70.4%, p<0.001), obesity (18.7% vs. 14.9%, p<0.001), peripheral vascular disorders (PVD) (17.9% vs. 13.4%, p<0.001), and chronic obstructive pulmonary disease (COPD) (22.8% vs. 16.8%, p<0.001) as compared to euthyroid patients in unmatched comparison (Table 1).

The propensity-matched (1:1) cohorts consisted of 827 (weighted N=4135) patients in both the groups. The proportions of age, sex, race, type of admission, location/teaching status of the hospital were comparable in both unmatched and propensity-matched cohorts. Similar to unmatched cohorts, hypertension, dyslipidemia, PVD, and COPD were more prevalent in the hypothyroid group after propensity matching (Table 1). However, the significant difference in the prevalence of diabetes with chronic complications between the two groups disappeared. In addition, the comorbidities uncomplicated diabetes (31.0% vs 27.8%, p=0.002) and coagulopathy (3.3% vs. 4.5%, p=0.004) were found to be more prevalent in the hypothyroid group after propensity score matching.

**Table 1:** Impact of Hypothyroidism on Outcomes of Percutaneous Coronary Intervention with Coronary Atherectomy for Calcified Coronary Lesions: Before vs. After Propensity-score Matched Analysis

Variables	Before Matching			Propensity-Matched Cohort (1:1)		
	Euthyroid	Hypothyroid	P-value	Euthyroid	Hypothyroid	P-value
Unweighted admission	8274	931		827	827	
Weighted admission	41370	4655		4135	4135	
Age in years (Mean±SD)	65±13	72±12	<0.001	46±9	72±12	<0.001
Indicator of Sex			<0.001			<0.001
Male	72.7%	45.4%		51.9%	44.6%	
Female	27.3%	54.6%		48.1%	55.4%	
Race			<0.001			<0.001
White	74.8%	82.5%		74.2%	83.8%	
African American	8.7%	4.9%		10.9%	4.4%	
Hispanic	8.2%	6.8%		9.1%	6.5%	
Asian and Pacific Islander	3.2%	2.3%		2.7%	1.7%	
Native American	0.6%	0.6%		0.7%	0.5%	
Others	4.6%	3.1%		2.4%	3.1%	
Type of Admission			0.001			<0.001
Non-elective	81.5%	79.5%		91.2%	81.0%	
Elective	18.5%	20.5%		8.8%	19.0%	
Location/Teaching Status of Hospital			0.108			0.007
Rural	3.6%	3.5%		3.7%	3.1%	
Urban - non teaching	32.8%	34.4%		38.8%	36.2%	
Urban - teaching	63.5%	62.1%		57.4%	60.7%	
Comorbidities						
Hypertension	72.2%	79.3%	<0.001	63.4%	78.8%	<0.001
Diabetes, uncomplicated	30.6%	30.1%	0.429	27.8%	31.0%	0.002
Diabetes with chronic complications	6.3%	12.0%	<0.001	11.1%	11.0%	0.861
Dyslipidemia	70.4%	73.4%	<0.001	67.0%	73.4%	<0.001
Obesity	14.9%	18.7%	<0.001	29.5%	18.5%	<0.001
Congestive heart failure	1.4%	1.5%	0.531	1.2%	1.5%	0.337
Coagulopathy	4.3%	4.5%	0.558	3.3%	4.5%	0.004
Peripheral vascular disorders	13.4%	17.9%	<0.001	6.3%	17.2%	<0.001
Smoking	43.4%	33.8%	<0.001	57.0%	33.9%	<0.001
Chronic obstructive pulmonary disease	16.8%	22.8%	<0.001	16.7%	23.3%	<0.001
Postoperative Outcomes						
All-cause in-hospital mortality	3.2%	4.1%	0.002	1.0%	4.1%	<0.001
Postoperative myocardial infarction	8.8%	9.7%	0.037	8.7%	9.8%	0.088
Any cardiac complications	12.4%	12.4%	0.887	10.5%	12.2%	0.015
Postoperative respiratory failure	0.9%	1.6%	<0.001	0.5%	1.7%	<0.001
Postoperative stroke	0.2%	0.3%	0.105	0.2%	0.4%	0.317
Acute kidney injury requiring dialysis	0.8%	1.3%	<0.001	0.6%	1.2%	0.004
Postoperative infection	1.8%	2.1%	0.154	1.3%	2.4%	<0.001
Length of stay (days) (Mean±SD)	4.2±5.9	4.8±5.3	<0.001	3.9±4.5	5.0±5.4	<0.001
Total hospital charges (USD) (Mean±SD)	107,711±103,242	116,379±97,690	<0.001	95,589±78,745	118,072±97,653	<0.001
P- value <0.05 indicates clinical significance.						

In unmatched comparison, primary outcomes including all-cause in-hospital mortality (4.1% vs. 3.2%, p<0.01) and postoperative myocardial infarction rate (9.7% vs. 8.8%, p<0.05) were higher in the hypothyroid group as compared to the euthyroid group. However, there was no statistically significant difference in the incidence of any cardiac complications and postoperative stroke between the two groups. In addition, the hypothyroid group appeared to have a higher rate of other complications such as postoperative respiratory failure (1.6% vs. 0.9%, p<0.001) and acute kidney injury (AKI) requiring dialysis (1.3% vs. 0.8%, p<0.001) as compared to the euthyroid group. Secondary outcomes including LOS (days) (4.8±5.3 vs. 4.2±5.9, p<0.001) and total hospital charges (mean $116,379 vs. $107,711, p<0.001) were also higher in the hypothyroid group (Table 1).

Similar to unmatched analysis, propensity-matched analysis demonstrated significantly higher in-hospital mortality (4.1% vs. 1.0%. p<0.001), postoperative respiratory failure (1.7% vs. 0.5%, p<0.001), AKI requiring dialysis (1.2%, 0.6%, p<0.001), postoperative infection (2.5% vs. 1.3%, p<0.001), LOS (5.0±5.4 vs. 3.9±4.5, p<0.001), and total hospital charges (118,072±97,653 vs. 95,589±78,745, p<0.001) in the hypothyroid group. Although numerically higher in the hypothyroid group, there was no statistically significant difference in postoperative myocardial infarction incidence (9.8% vs. 8.7%, p=0.088) between the propensity-matched cohorts. Contrary to unmatched assessment, matched analysis revealed a higher incidence of any cardiac complications (12.2% vs. 10.5%, p=0.015) and postoperative infection (2.4% vs. 1.3%, p<0.001) in hypothyroid patients.

## Discussion

Propensity-matched analysis demonstrated that the hypothyroid state is associated with higher risk of cardiovascular comorbidities, any cardiac complications, and all-cause in-hospital mortality, which is consistent with the previously published studies showing the negative impact of subclinical and clinical hypothyroidism on PCI outcomes [4,5]. With a major strength of being performed on the largest inpatient sample in the US, our study also has a few potential limitations. As with any large database, there is a scope of administrative coding errors. Since the database does not provide the exact number of thyroid hormone levels, it is not possible to assess the strength of association for causality and establish the association between subclinical hypothyroidism and the worse outcomes.

## Conclusions

In conclusion, by using propensity-matched analysis in the largest US database, we discovered that hypothyroidism was significantly associated to higher cardiovascular comorbidities, LOS, total hospital charges, cardiovascular complications, postoperative respiratory failure, AKI requiring dialysis, postoperative infection, and all-cause in-hospital mortality in the patients undergoing PCI with CA. Therefore, it is recommended that health care professionals be extra vigilant for the development of these complications in patients with hypothyroidism undergoing PCI with CA. Categorization of these patients as “high-risk” may help in the early recognition and management of these complications and curb higher cardiovascular morbidity and mortality in this population.
